# Zein-Derived Peptides from Corn Promote the Proliferation of C_2_C_12_ Myoblasts via Crosstalk of mTORC1 and mTORC2 Signaling Pathways

**DOI:** 10.3390/foods13060919

**Published:** 2024-03-18

**Authors:** Mohammad Sadiq Amin, Binbin Yu, Dongjing Wu, Yujia Lu, Wei Wu, Jing Wang, Yuhao Zhang, Yu Fu

**Affiliations:** 1 College of Food Science, Southwest University, Chongqing 400715, China; sadiqamin1112@gmail.com (M.S.A.); yubinbin@email.swu.edu.cn (B.Y.); a22252225wdj@163.com (D.W.); zhy1203@swu.edu.cn (Y.Z.); 2 Chongqing Key Laboratory of Speciality Food Co-Built by Sichuan and Chongqing, Chongqing 400715, China; 3 Department of Storage and Processing of Animal Products, Faculty of Animal Science, Afghanistan National Agricultural Sciences and Technology University, Kandahar 3801, Afghanistan; 4 Department of Epidemiology, T.H. Chan School of Public Health, Harvard University, Boston, MA 02115, USA; yujia_lu@hsph.harvard.edu; 5 College of Animal Science and Technology, Southwest University, Chongqing 400715, China; weiwu2019@swu.edu.cn; 6 China-Canada Joint Lab of Food Nutrition and Health (Beijing), Beijing Technology & Business University (BTBU), Beijing 100048, China; wangjing@th.btbu.edu.cn

**Keywords:** myoblast, zein peptides, cell proliferation, cell cycle, anti-apoptotic, molecular mechanism, mTOR pathway

## Abstract

Dietary protein supplementation has emerged as a promising strategy in combating sarcopenia. Furthermore, searching for alternatives of animal proteins has been a hot topic. The present study aimed to investigate the effects of zein peptides on C_2_C_12_ myoblasts and explore their potential molecular mechanisms. The proliferative, cell cycle, and anti-apoptotic activities of zein peptides were evaluated. Peptidomics analysis and transcriptome sequencing were employed to explore the structure-activity relationship and underlying molecular mechanisms. The results indicated that zein peptides (0.05–0.2 mg/mL) exerted a significant proliferation-promoting impact on C_2_C_12_ cells, via increasing cell viability by 33.37 to 42.39%. Furthermore, zein peptides significantly increased S phase proportion and decreased the apoptosis rate from 34.08% (model group) to 28.96% in C_2_C_12_ cells. In addition, zein peptides exhibited a pronounced anti-apoptotic effect on C_2_C_12_ cells. Zein peptides are abundant in branch-chain amino acids, especially leucine. Transcriptomics analysis revealed that zein peptides can promote proliferation, accelerate cell cycle, and improve protein synthesis of muscle cells through mTORC1 and mTORC2 signaling pathways.

## 1. Introduction

The aging population has emerged as a significant global social concern, primarily augmenting the burden of social welfare and the prevalence of diverse diseases in the elderly [1]. Sarcopenia is frequently observed, which is characterized by a depletion of muscle mass, strength, and/or physical capability. It is estimated that individuals (5–13%) aged over 60 normally experience low skeletal muscle mass and weakened function, resulting in an increased risk of injuries, such as frailty and falls [2]. Hence, sarcopenia not only impacts the daily lives of elderly individuals, but also presents significant challenges for families and society. Hence, it is of great significance to investigate strategies to prevent and alleviate sarcopenia.

During the development process of skeletal muscle, the synthesis and degradation of skeletal muscle proteins contribute to maintaining muscle balance [3]. However, in the elderly, muscle protein synthesis is slowed down, while degradation is increased, leading to decreased muscle mass and thus inducing sarcopenia [4]. Sarcopenia is closely related to major signaling pathways, including phosphatidylinositol 3-kinase (PI3K), protein kinase B (PKB)/AKT, mammalian target of rapamycin (mTOR), and ubiquitin-proteasome system (UPS). Several factors, such as amino acids, peptides, insulin, or energy, stimulate protein synthesis primarily through the mTOR signaling pathway [5]. Peptides containing branched-chain amino acids (BCAA) are implicated in involving mTOR mediators, which include the phosphorylation of downstream targets p70S6K and 4E-BP1, binding of eIF4E to eIF4G, and ribosomal protein S6. These effects subsequently promote protein synthesis, while inhibiting protein degradation by attenuating forkhead transcription factor (FoxO) and reducing proteasome activity [6].

Food proteins can serve as a potential dietary supplement for alleviating sarcopenia [7]. Daytime and nighttime supplements of casein and whey protein can increase muscle protein synthesis [8]. In addition to proteins, food protein-derived peptides have also demonstrated the potential to alleviate sarcopenia. Protein hydrolysates containing dipeptides and tripeptides are more readily absorbed than free amino acids and intact proteins, thereby enhancing their efficacy [9]. Furthermore, compared to protein supplements, bioactive peptides exhibit higher biocompatibility and lower immunogenicity [10]. It has been shown that soluble whey protein hydroxylate (WP-S) can stimulate the PI3K/Akt/mTOR signaling pathways while suppressing UPS, ultimately resulting in increased muscle protein content. This regulatory effect on protein synthesis and degradation suggests the potential of WP-S to effectively alleviate sarcopenia. Collagen dipeptide (Hyp-Gly) has been reported to promote myogenic differentiation and myotube hypertrophy in C_2_C_12_ myoblasts, increasing myotube size and specific protein expression [11]. Consequently, bioactive peptides are promising as dietary supplements for alleviating sarcopenia.

A number of animal-derived bioactive peptides are promising in alleviating sarcopenia, while plant-derived bioactive peptides have been reported to exhibit anti-sarcopenic effects [12], including rice-derived bran peptides and potato peptides. Zein, a protein by-product of corn starch production in the food and brewing industries, can used as a skeleton protein in the production of cultivated meat [13]. Furthermore, it can be enzymatically hydrolyzed and ultrafiltrated to produce zein peptides. These peptides are rich in BCAA, which are comparable to the content found in whey protein. Therefore, it is hypothesized that zein peptides may serve as a valuable nutritional supplement with beneficial effects on muscle cells. However, there is a dearth of studies on the effects of zein peptides on myoblasts and their molecular mechanisms. In this regard, this study explored the effects of zein peptides on cell proliferation, differentiation, and apoptosis in myoblast C_2_C_12_ cells. Peptidomics analysis was conducted on zein peptide sequences to explore their structure-activity relationship. Furthermore, the molecular mechanism underlying was elucidated through transcriptomics analysis.

## 2. Materials and Methods

### 2.1. Materials

Zein (protein content, 90.2%) was obtained from Yuanye Biotechnology Co., Ltd. (Shanghai, China). The myoblast C_2_C_12_ cell line was donated by Chongqing Medical University (Chongqing, China). Fetal bovine serum, Dulbecco’s modified eagle’s medium (DMEM) with double antibody, 0.25% trypsin, dimethyl sulfoxide (DMSO), phosphate-buffered solution (PBS), and recombinant mouse LR3 IGF-1/Long R3 IGF-1 were purchased from Saimike Biotechnology Co., Ltd. (Chongqing, China). Recombinant murine TNF-α/TNFSF2 and cell counting kit-8 (CCK-8) were purchased from Beyotime Biotechnology Co., Ltd. (Shanghai, China). Propidium iodide (PI) and RNase A were obtained from Sigma-Aldrich (St. Louis, MO, USA). Annexin V-FITC was purchased from Elabscience Biotechnology Co., Ltd. (Wuhan, China). The primary antibodies were obtained from Cell Signaling Technology (Shanghai, China), including rabbit anti-PI3K, rabbit anti-phospho-PI3K, rabbit anti-AKT antibody, rabbit anti-phospho-AKT, rabbit anti-mTOR antibody, rabbit anti-phospho-mTOR, rabbit anti-eif4E, and rabbit anti-PKC. The secondary antibody (HRP-labeled goat anti-rabbit IgG antibody) and GAPDH were purchased from Bioss Biotechnology Co., Ltd. (Beijing, China).

### 2.2. Preparation of Zein Peptides

Zein was dispersed using Milli-Q water at the concentration of 5% (*w*/*w*) and adjusted to pH 8.0 using NaOH (2 mol/L). Alcalase was selected for the subsequent enzymatic hydrolysis due to its high hydrolytic efficiency to release bioactive peptide sequences. Alcalase (1%, *w*/*w*) was added to the protein mixture to initiate enzymatic hydrolysis process with continuous stirring at 60 °C. After hydrolysis for 6 h, enzymatic hydrolysis was terminated by heating the hydrolysate solution for 20 min in boiling water to inactivate the enzyme. Protein hydrolysate was further cooled to room temperature. By centrifugation at 8000× *g* for 10 min at 25 °C, the supernatant was further recovered. Subsequently, protein hydrolysates (supernatant) were ultrafiltered using 1 kDa molecular weight cut-off centrifugal ultrafiltration device (Millipore, Bedford, MA, USA). The peptide fraction (<1 kDa) was collected, concentrated, and then freeze-dried for further use. Peptide fraction (below 1 kDa) was selected owing to its high bioaccessibility and bioactivity.

### 2.3. Peptidomics Analysis of Zein Peptides Using LC-MS/MS Analysis

Zein peptides were analyzed using LC-MS/MS using EASY-nLC™ 1200 System with a C18 column (Acclaim PepMap-C18, 75 μm × 15 cm, 3 μm, 100 Å) coupled with an Orbitrap Mass Spectrometer (Thermo Fisher Scientific, Saint Louis, MO, USA). The injection volume of each sample was 10 μL. The flow rate was fixed at 200 nL/min. Buffer A was 0.1% formic acid (FA), while buffer B was 0.1% FA in 80% acetonitrile. The injection volume of each sample was 10 μL. The flow rate was fixed at 0.25 mL/min and the gradient consisted of 100% buffer A for 5 min, followed by a linear increase from 0% to 45% buffer B at 45 min On-line MS/MS spectra were recorded using first Full MS method with positive mode, 70,000 resolution, AGC target of 3 × 10^6^ and max IT 50 ms. The top 10 spectra were analyzed using the dd-MS^2^ method with resolution of 35,000 and automatic gain control (AGC) target of 1 × 10^6^. The raw MS files were further processed using MaxQuant version 1.6.10, and MS/MS spectra were searched against *Zea mays* (Maize) proteome from the Swiss-Prot database.

### 2.4. Cell Culture

Owing to the inherent challenges associated with obtaining primary cells, the C_2_C_12_ cell line, derived from murine myoblasts, is frequently employed in research pertaining to muscle cell research. The myoblast C_2_C_12_ cells were cultured in T-25 cm^2^ cell culture flasks with media composed of 5 mL of DMEM supplemented with 10% (*v*/*v*) fetal bovine serum, 100 U/mL penicillin, and 100 µg/mL streptomycin. Cells were maintained at 37 °C with 5% CO_2_ and 95% relative humidity. Once the cells reached 90% confluence, cell medium was changed every other day. To examine the effects of zein peptides on the growth of C_2_C_12_ cells, the appropriate amounts of zein peptides (0.01, 0.02, 0.05, 0.1, and 0.2 mg/mL) were added to media.

### 2.5. Effects of Zein Peptides on Proliferation of C_2_C_12_ Cells

C_2_C_12_ cells were seeded onto 96-cell plates and cultured at a density of 2–5 × 10^3^ cells in 50 μL of growth medium per well for 3 h. Then, zein peptides (0.01, 0.02, 0.05, 0.1, and 0.2 mg/mL), IGF-1 (100 ng/mL) as the positive control group, and growth medium were added into the wells and cultured for 24 and 48 h, respectively. The cells without addition of zein peptides were regarded as the negative control. Subsequently, CCK-8 (10 μL) was added to each well and incubated for another 1.5 h. The absorbance was subsequently measured at the wavelength of 450 nm using a SpectraMax i3 multiplate microplate reader [14]. The absorbance of each well was recorded, and cell viability was calculated by the following equation:

(1) $$\mathrm{Cell}\;\mathrm{viability}\;\left(\%\right)=\frac{\mathrm{OD}\;\mathrm{value}\;\mathrm{of}\;\mathrm{treatment}\;\mathrm{groups}\;-\;\mathrm{OD}\;\mathrm{value}\;\mathrm{of}\;\mathrm{blank}\;\mathrm{groups}}{\mathrm{OD}\;\mathrm{value}\;\mathrm{of}\;\mathrm{negative}\;\mathrm{groups}\;-\;\mathrm{OD}\;\mathrm{value}\;\mathrm{of}\;\mathrm{blank}\;\mathrm{groups}}\times 100,$$

where treatment group was zein peptides (0.01, 0.02, 0.05, 0.1, and 0.2 mg/mL) and IGF-1 (100 ng/mL) was used as the positive control.

### 2.6. Impacts of Zein Peptides on the Cell Cycle of C_2_C_12_ Cells

C_2_C_12_ cells were seeded at the concentration of 4.5–5 × 10^5^ cells per well in 6-well plates in growth medium for 3 h. Then, the growth medium was replaced with serum-free DMEM, and cells were starved for 16 h to synchronize to G_0_ phase. Subsequently, the medium was replaced with growth medium with or without zein peptides (0.2 mg/mL) using 6-well plates. After 24 h incubation, cells were harvested and fixed with 70% ice-cold ethanol at 4 °C overnight, rinsed with PBS, and finally stained with PI and RNase A for 30 min at room temperature in the dark. CytoFLEX flow cytometer was used for cell cycle analysis (405/488 nm lasers) (Beckman Coulter, Brea, CA, USA), and data were analyzed using ModFit software (Version 5.0.9).

### 2.7. Impacts of Zein Peptides on Anti-Apoptotic Effect of C_2_C_12_ Cells

*Prevention treatment.* C_2_C_12_ cells at the concentration of 3.5 × 10^5^ cells per well were first cultured in 6-well plates for 3 h. The cells were further treated with or without zein peptides (0.2 mg/mL). After 24 h incubation, the growth medium of each well was replaced with a fresh growth medium with or without 100 ng/mL TNF-α (blank groups) and incubated for 24 h.

*Therapeutical treatment.* C_2_C_12_ cells were seeded in 6-well plates at around 3.5 × 10^5^ cells/well. After 3 h culture, the cells were treated with TNF-α (100 ng/mL) for 24 h. Subsequently, either zein peptides (0.2 mg/mL) or equivalent amounts of growth medium were added to the plate. The cells were harvested after 24 h and detected according to the procedures described above. After being harvested, the cells were treated with binding buffer (500 μL) after washing twice with PBS. Finally, the cells were incubated with Annexin V-FITC and PI at room temperature for 10 min. Apoptosis analysis was performed using a CytoFLEX flow cytometer (Beckman Coulter, 405/488 nm lasers), and the data were subsequently analyzed using FlowJo v10.0.8 software (Flowjo, Treestar Inc., Ashland, OR, USA).

### 2.8. Extraction of RNA

The T25 culture flasks covered with 95% or more cells were subjected to digestion, then 5 mL of growth medium was added to resuspend cells and collected in centrifuge tubes (15 mL). Thereafter, cells were counted on a cell counting plate. Cells at the concentration of 3.5–4 × 10^6^ were seeded onto T25 with fresh growth medium. After 3 h culture for cell adhesion, zein peptide solution (2 mg/mL) of 2.5 mL and the filtered growth medium (negative control group) were added. After 24 h culture, the growth medium in T25 was discarded. Subsequently, the pre-cooled PBS solution of 3 mL was slowly added along the side wall and shaken gently for a few seconds. After PBS was discarded, the cells were washed once again. Then, cells were added to 1 mL of TRIzol for every 5 × 10^6^ cells and placed at 4 °C for 10 min. Afterwards, cell suspension (1 mL) was collected in each pre-cooled RNase-free cryopreservation tube and frozen in liquid nitrogen.

### 2.9. Transcriptomics Analysis

Transcriptomics analysis was performed on the Illumina NovaSeq 6000 sequencing platform for all extracted mRNAs. Library construction was performed using the Truseq TM RNA sample prep kit method, and sequencing was performed on Illumina NovaSeq 6000 sequencing platform after library normalization, purity assessment and sequencing control. The offline sequencing data were subjected to quality control and sequence comparison, and then mRNA annotation information for samples was obtained. The obtained data were analyzed through the free online platform of Majorbio Cloud Platform (www.majorbio.com, accessed on 5 June 2022). The mRNAs were further counted for expression using RSEM (v1.3.3) and subjected to TPM homogenization. Differential expression analysis was performed using the R language DESeq2 package, setting parameters *p* < 0.05 and up/down-regulated difference multiples ≥2 (i.e., |Log2FC| ≥ 1). Differential miRNA target genes were predicted using miRWalk online software (http://mirwalk.umm.uni-heidelberg.de/search_mirnas/, accessed on 5 June 2022) and the R language VennDiagram package to perform GO and KEGG enrichment analysis after the intersection of the two genes; Cytoscape v3.6.1 software was used to draw mRNA visualization network diagrams.

### 2.10. Statistical Analysis

The data are presented as mean ± standard deviation from at least three independent experiments. Results were analyzed using a one-way ANOVA and Duncan post hoc analysis by SPSS 26.0. *p* < 0.05 was considered statistically significant.

## 3. Results

### 3.1. Impacts of Zein Peptides on the Proliferation of C_2_C_12_ Cells

**Figure 1:**
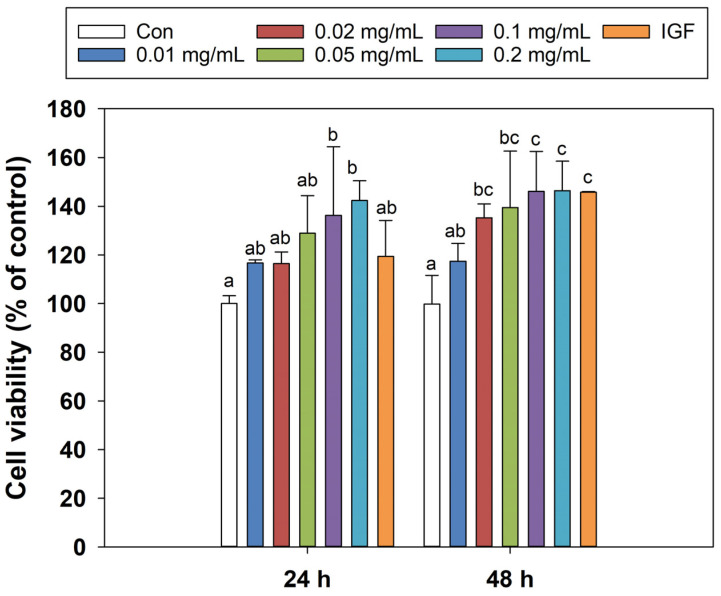
Effects of zein peptides on proliferation of C_2_C_12_ cells. Different letters above the columns indicate that the means of different groups were significantly different at 24 or 48 h (*p* < 0.05).

As shown in Figure 1, compared with the negative control group, zein peptides at concentrations of 0.05–0.2 mg/mL exhibited a significant proliferation-promoting effect on C_2_C_12_ cells after 24 h-incubation, and the cell viability was increased by 33.37–42.39% (*p* < 0.05). The cell viability of zein peptides (0.2 mg/mL) was the highest, which was higher than that of the positive control group (142.39% vs. 119.41%). After incubation for 48 h, the cell viability increased at all tested concentrations of zein peptides, especially the concentrations of 0.02–0.2 mg/mL demonstrating significant proliferation-promoting effects. Compared with the negative control group, and the cell viability was increased by 37.74–46.34%, showing a dose-concentration effect. Notably, zein peptides (0.2 mg/mL) exhibited the strongest proliferation-promoting effect, which was superior to the positive control group, with cell viability of 146.34% and 145.74%, respectively. Although the cell viability (146.34%) after 48 h treatment with zein peptides (0.2 mg/mL) was slightly increased compared to cell viability (142.39%) after 24 h, there was no significant difference (*p* > 0.05). Therefore, in the subsequent treatment, the concentration of zein peptides was fixed at 0.2 mg/mL for 24 h incubation.

### 3.2. Impacts of Zein Peptides on the Cycle of C_2_C_12_ Cells

**Figure 2:**
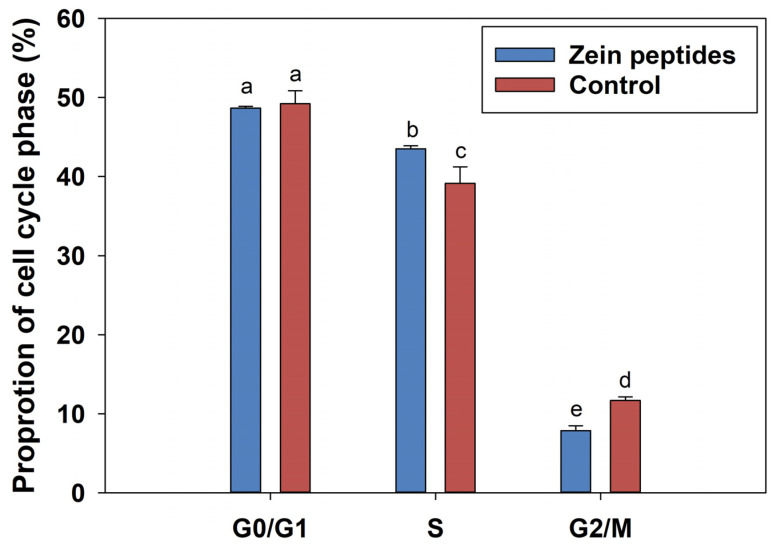
Effects of zein peptides on cell cycle of C_2_C_12_ cells analyzed using flow cytometry and the percentage of cell population distribution in G_0_/G_1_, S, or G_2_/_M_. Different letters above the columns indicate that the means of different groups were significantly different (*p* < 0.05).

The impact of zein peptides (0.2 mg/mL) on the cell cycle progression of C_2_C_12_ cells was investigated using flow cytometry after 24 h incubation. The results of flow cytometric are illustrated in Figure 2. The highest peak represents the G_0_/G_1_ phase, which indicates the diploid (2N) chromosome, while the lower peak represents the G_2_/_M_ phase, which indicates the tetraploid (4N) chromosome. The S phase is between these two peaks. According to Figure 2, the percentage of S-phase in C_2_C_12_ cells was significantly increased from 39.13% to 43.52% after 24 h treatment with 0.2 mg/mL zein peptides (*p* < 0.05), while the percentage of G_0_/G_1_ phase cells was decreased from 49.20% to 48.65%. The results indicated that zein peptides could significantly increase the cell proportion of the S phase in C_2_C_12_ cells and promote the accelerated transition from diploid to tetraploid (S phase), which was consistent with the results of cell proliferation that zein peptides can promote the proliferation in C_2_C_12_ cells by accelerating cell cycle progression.

### 3.3. Impacts of Zein Peptides on the Apoptosis of C_2_C_12_ Cells

**Figure 3:**
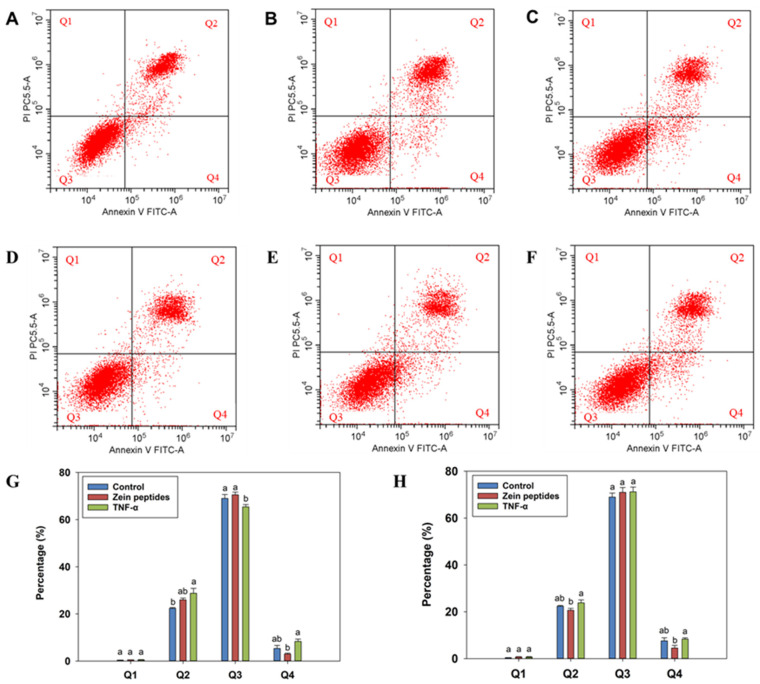
Effects of zein peptides on the apoptosis of C_2_C_12_ cells. Q1 represents necrotic cells; Q2, late apoptotic cells; Q3, live cells; Q4, early apoptotic cells; and the apoptosis rate is the ratio of early and late apoptotic cells (Q2 + Q4). (**A**–**C**) denote zein peptides, model, and control in the prevention group, respectively. (**D**–**F**) denote zein peptides, model, and control in the treatment group, respectively. (**G**,**H**) represent cell distribution percentage (%) of prevention group and treatment group, respectively. Different letters above the columns indicate that the means of different groups were significantly different (*p* < 0.05).

The apoptotic results of flow cytometry are shown in Figure 3. Cell apoptosis is measured using FITC–annexin V double-stained flow cytometry. Q1, the dead cells; Q2, the later apoptotic cells; Q3, the viable cells; Q4, the early apoptotic cells. Figure 3A–C denote zein peptides, model, and control in the prevention group, while Figure 3D–F denote zein peptides, model, and control in the treatment group. Figure 3G,H represent cell distribution percentage (%) of prevention group and treatment group, respectively. In the prevention group, the apoptosis rate in the model group (34.08%) was remarkably higher than that in the control group (29.48%), indicating that TNF-α could induce apoptosis in cells. Zein peptides (0.2 mg/mL) could reduce the apoptosis rate from 34.08% (model group) to 28.96% (*p* < 0.05). By contrast, in the treatment group, the apoptosis rate of zein peptide group (26.95%) was lower than that in the model group (29.47%). Meanwhile, 24 h treatment with zein peptides increased the percentage of live cells from 69.76% to 72.43%, which was higher than that in the control group (70.18%). The above-mentioned phenomenon indicated that zein peptides (0.2 mg/mL) can prevent and alleviate the apoptosis of C_2_C_12_ cells.

### 3.4. Identification of Peptide Sequences using Peptidomics Analysis

Further analysis of peptide sequence can provide a reference for the structure-function of zein peptides. Peptidomics analysis with the aid of LC-MS/MS analysis was performed, and a total of 72 peptide sequences were identified. The top 20 peptide sequences were selected based on peptide abundance and blast analysis (Table S1). It was found that zein peptides are abundant in Leu, Pro, and Ser, while the ratio of branched-chain amino acids (Leu, Ile and Val) was as high as 30.23%. Several peptides were abundant in Leu, including LLPPYLPS, SLLPPYLPS, SLLPPYLSPA, LLPPYLSPA, RQQLLNPL, QRQQLLNPL, LLPPYLSP, and others.

### 3.5. Transcriptomics Analysis

In order to further understand the underlying molecular mechanism of zein peptides responsible for the beneficial effects on C_2_C_12_ cells, transcriptomic sequencing technology was performed to analyze the differentially expressed genes (DEGs). Moreover, cluster, GO, and KEGG analyses were performed based on DEGs.

#### 3.5.1. DEGs and Cluster Analysis

**Figure 4:**
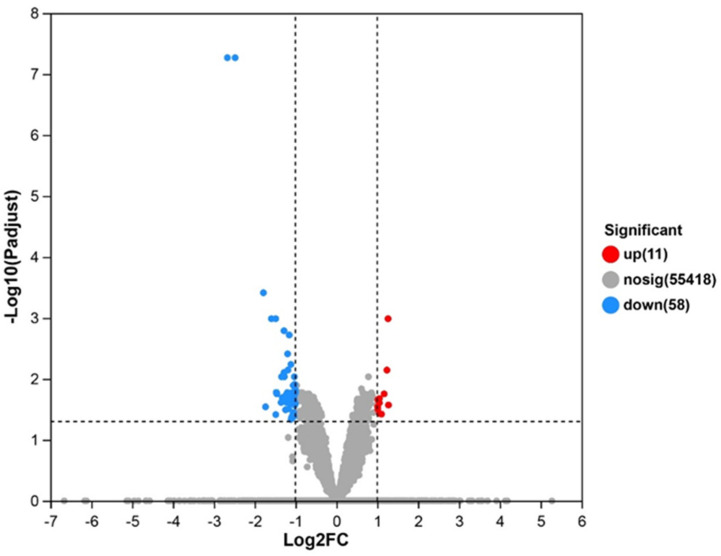
Volcano diagram of differentially expressed genes from zein peptides-treated group and control group. The genes with significantly up-regulated expression are in red, the genes with significantly down-regulated expression are in blue, and the genes with no significantly differential expression are in gray.

The transcriptome sequencing analysis was performed on RNA extracted from C_2_C_12_ cells treated with zein peptides. The volcano plot of DEGs is shown in Figure 4. We found 69 DEGs in the zein peptide treatment group and the control group, among which 11 genes were up-regulated and 58 genes were down-regulated.

Cluster analysis (k-means clustering, k = 10) was performed on the DEGs in mTOR pathway after zein peptide treatment, as illustrated in heatmap of Figure S1. In general, genes can be mainly divided into two categories, namely up-regulated and down-regulated genes. It has been shown that gene abundance was enriched in mTOR signaling pathway and the DEGs (23) present in the mTOR pathway were analyzed.

#### 3.5.2. GO Analysis

GO analysis was further used to identify the up-regulated and down-regulated genes, which can provide a comprehensive description of genes and gene properties, describing gene and protein functions across three main areas, namely biological processes, cellular components, and molecular functions. In C_2_C_12_ cells treated with zein peptides, Figure S2A,B illustrate the GO annotation and functional enrichment of down-regulated expressed genes. As can be seen from Figure S2A, the biological processes involved in down-regulating the expression of genes mainly include multi-organism processes, metabolic processes, cellular component organization or biogenesis, localization, development processes, response to stimuli, biological regulation, and cellular processes; cell components mainly include supramolecular complexes, synapses, membranes, organelles, cell parts, and protein-containing complexes; molecular functions include catalytic activity, molecular function regulation and binding. In Figure S2B, the GO function enrichment shows that down-regulated expression genes are mainly related to amino acid stimulation, signal transduction, response to endogenous stimulation, response to stimuli, signal regulation, cell communication, signal regulation, protein kinase activity, protein phosphorylation, and kinase activity regulation.

#### 3.5.3. KEGG Analysis

KEGG can systematically analyze the metabolic pathways of genes in cells, revealing the corresponding metabolic pathways, which can contribute to the elucidation of relevant molecular mechanisms. KEGG analysis was conducted on the target gene to analyze the enrichment pathways in top 20. In Figure S3A, the KEGG function enrichment of up-regulated genes shows that mTOR, FoxO, insulin, and PI3K/Akt signaling are among the pathways involved in up-regulating gene expression. In addition to growth hormone synthesis, secretion, and function, mitogen-activated protein kinase (MAPK) and Wnt signaling pathways play an important role in protein synthesis. As shown in Figure S3B, the down-regulated genes mainly involve signaling pathways, such as mTOR, stem cell pluripotency, insulin, autophagy, and cell aging.

### 3.6. The Potential Signaling Pathway

**Figure 5:**
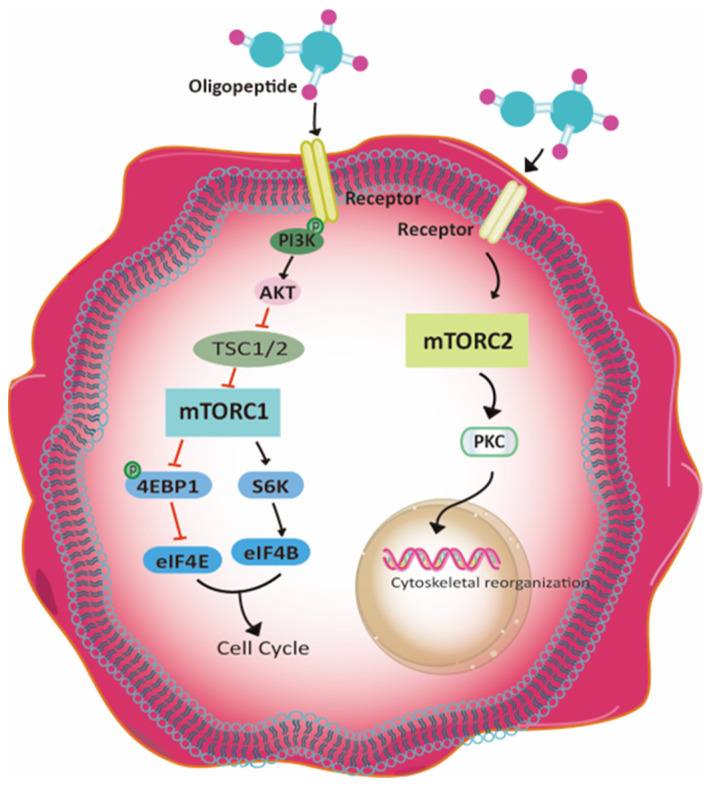
The proposed signaling pathway in zein peptides-treated C_2_C_12_ cells.

According to the analysis data from the RNA-Seq data, the proteins involved in mTORC1 and mTORC2 signaling pathways in C_2_C_12_ cells treated with zein peptides were altered, among which the protein expression of PI3K, p-PI3K, AKT, p-AKT, mTORC2, and PKC were up-regulated, while the protein expression of mTORC2 and eif4E were down-regulated. Hence, the proposed signaling pathway responsible for zein peptides-treated C_2_C_12_ cells is illustrated in Figure 5. The involved signaling pathway is mainly related to cell proliferation, cell cycle and protein synthesis. The down-regulated proteins in mTORC1 signaling pathway could accelerate the cell cycle, further promoting cell proliferation. In addition, up-regulation of mTORC2 signaling pathway can promote PKC expression, thereby activating the regulation of actin cytoskeleton as well as protein synthesis in muscle cells.

## 4. Discussion

### 4.1. Effect of Zein Peptides on the Cell Proliferation

The proliferation of muscle cells is an important process in the development of skeletal muscle. C_2_C_12_ cell line is the most commonly used mature muscle cell line for muscle study [15]. It has been reported that collagen dipeptide (Hyp-Gly) can promote the proliferation and differentiation of murine myogenic C_2_C_12_ cells [15]. Recently, bovine collagen peptide (GDAGPPGPAGPAGPPGPIG) have been reported to exhibit a significant impact on the proliferation of C_2_C_12_ cells (proliferation rate, 18.5%, *p* < 0.05) [16]. Furthermore, potato protein hydrolysate could enhance proliferation and myogenic differentiation in C_2_C_12_ cells, which further increased myogenic protein synthesis and prevented its degradation [13]. In this study, zein peptides at 0.05–0.2 mg/mL for 24 h had a significant proliferation-promoting effect on C_2_C_12_ cells, which demonstrated the great potential of zein peptides to promote cell proliferation of myoblasts.

### 4.2. Effect of Zein Peptides on Cell Cycle

Cell cycle can determine the ratio of cell proliferation, while the increased rate of cell proliferation is mainly due to the accelerated cell cycle process. It was reported that after 16 h treatment with GDAGPPGPAGPAGPPGPIG, the S-phase of C_2_C_12_ cells displayed an upregulation of 7%, which promoted cell proliferation. Additionally, the specific peptide could increase the mRNA expression of Cyclin A and Cyclin E, which are essential for cell cycle progression [17]. PEDF-derived peptide has been reported to enhance proliferation and promote cyclin D1 expression in rat muscle fiber primary satellite cells [18]. In this study, the proportion of C_2_C_12_ cells (DNA synthesis phase) increased significantly (*p* < 0.05), and the proportion of G_0_/G_1_ cells decreased after 24 h treatment with zein peptides (0.2 mg/mL). The present result confirmed that zein peptides can stimulate DNA synthesis in C_2_C_12_ cells, promote cell cycle transition from G_0_/G_1_ phase to S phase, and accelerate cell cycle progress.

### 4.3. Effect of Zein Peptides on Anti-Apoptotic Effect of C_2_C_12_ Cells

During muscle cell differentiation and development, cells die actively according to their specific procedures and are eliminated in the form of apoptotic cells, which is of great significance in muscle tissue repair [17]. Recently, it has been shown that the apoptosis rate in C_2_C_12_ cells was reduced by 4% and the late apoptotic rate by 5% after treatment of GDAGPPGPAGPAGPPGPIG, indicating that peptides can inhibit late apoptosis and promote the growth of C_2_C_12_ cells [17]. To the best of our knowledge, the current study for the first time reported the anti-apoptotic effect of zein peptides on myoblasts. Namely, zein peptides (0.2 mg/mL) treatment had a preventive and alleviative effect on apoptosis in C_2_C_12_ cells.

### 4.4. Analysis of Zein Peptide Sequences Using LC-MS/MS

It has been revealed in numerous clinical studies that Leu was a critical factor in stimulating muscle protein anabolism. In a previous study, subjects supplemented with either WPI (25 g) or milk protein (10 g), a blend of milk protein concentrates (95%), milk protein isolates (5%), and 3 g of total Leu, were shown to maintain skeletal muscle protein synthesis and improve muscle loss. When measured in comparison with total protein, a higher concentration of Leu promoted myofibrillar protein synthesis [19,20]. Recently, potato peptide hydrolysate (PPH902) has shown a cytoprotective effect on skeletal muscle cells by inducing phosphorylation of Akt and mTOR and promoting myoblast differentiation of C_2_C_12_ cells. Subsequently, DI-10, a decapeptide from PPH, has been studied in vitro for its effects on myoblast differentiation, muscle protein synthesis, and mitochondrial biogenesis. DI-10 treatment of C_2_C_12_ myoblasts accelerated the phosphorylation of kinases (ERK, AKT, and mTOR) in a dose-dependent manner [13]. DI-10 also increased phosphorylation of AKT, mTOR, and mitochondria-related transcription factors AMPK and PGC1α expression. Zein peptides in this study were abundant in Leu with a high proportion of BCAAs of 30.23% (top 20 abundant peptides), which was higher than that of a bio-whey concentrate (2.324 g/100 g) and whey protein isolate (28.937 g/100 g) [21]. As a result, zein peptides, with a high amount of Leu and BCAAs, can significantly promote the proliferation of C_2_C_12_ cells and exhibit a preventive and alleviating effect on cell apoptosis.

### 4.5. The Potential Molecular Mechanisms

The growth of myoblast is mediated by cell signaling pathways along the cell surface in response to extracellular signals, such as amino acids, peptides, hormones, cytokines, and mechanical damage [22]. mTOR and AMPK pathways play a crucial role in epigenetic regulation of myoblast differentiation [23]. In the present study, through transcriptomic analysis, 69 reported DEGs were found between zein peptides-treated group and the control group, of which 11 genes were up-regulated and 58 genes were down-regulated. The results indicated that after treatment with zein peptides, many biological processes and signaling pathways in muscle cells may undergo significant changes.

Through GO analysis of DEGs, it was found that the up-regulated DEGs mainly occurred in the nucleus and organelles that were involved in protein complexes, including biological processes, such as organization of cellular components or biogenesis, cellular processes [24]. As an upstream stimulator of mTOR, insulin plays an important role. It has been shown that dietary-induced insulin can independently stimulate muscle protein synthesis [25]. After binding to specific receptors, insulin can directly stimulate the activation of PI3K through phosphorylation, which then activates protein kinase B (PKB or Akt) [26], thereby activating mTORC1 and enhancing protein synthesis. In addition, the positive regulation of glucose transmembrane transport is also beneficial for protein synthesis. Glucose could stimulate the mTORC1 pathway by activating AMPK and thus promoting muscle protein synthesis [27]. Overall, the up-regulated genes showed that zein peptides promoted protein synthesis in C_2_C_12_ cells. Similarly, the down-regulated DEGs mainly occur in synapses, organelles, cell parts, and protein-containing complexes, including cellular components, tissues, biogenesis, response to stimulation, biological regulation, and other biological processes. The down-regulated DEGs further participate in the regulation of signal transduction, cellular response to stimuli, protein kinase activity, protein phosphorylation, etc. [28].

According to KEGG analysis, the up-regulated genes are mainly involved in AMPK, Wnt, insulin, MAPK, and PI3K-Akt signaling pathways. MAPK signaling pathway plays an important role in complex cellular processes, such as proliferation, differentiation, and survival. While the activation of the MAPK signaling pathway is associated with the differentiation ability of various stem cells [17], two main types of MAPK pathways include p38 MAPK and ERK1/2 [29]. Among them, p38 MAPK pathway mainly controls satellite cells, which is closely related to cell cycle. It has also been shown that p38 MAPK pathway can induce cell cycle and muscle-specific gene expression [30]. However, excessive expression of p38 signaling pathway can lead to disruption of asymmetric division in satellite cells, a decrease in self-renewal of satellite cells and inhibition of myoblast differentiation [31]. The MAPK pathway is also a major signal transduction cascade that ultimately triggers various biological cell responses to IGF-I [32]. By contrast, low-carboxy osteocalcin could induce the proliferation of C_2_C_12_ myoblasts through activation of PI3K/Akt and P38 MAPK pathways and promoted differentiation through activation of ERK1/2 pathway [33]. According to a recent study, potato hydrolysate DI-10 can accelerate the phosphorylation of promyelinating kinase in a dose-dependent manner, such as ERK, Akt, and mTOR in C_2_C_12_ myogenic cells, thereby promoting myogenic differentiation [13].

mTOR signaling pathway is also an important signaling pathway for cell survival and growth [34]. After treatment with zein peptides, up-regulation of mTOR/PI3K/Akt signaling pathways was observed. After mTORC1 activation, S6K (ribosomal protein S6 kinase beta) can be activated through phosphorylation to inhibit ATG1 (serine/threonine protein kinase ULK1) and 4E-BP (eukaryotic translation initiation factor 4E binding protein 1). ATG1 is associated with autophagy, and mTORC1 inhibits cell autophagy by inhibiting ATG1 [35]. 4E-BP is an inhibitor of eIF4E, which is related to protein synthesis, and mTORC1 inhibition of 4E-BP can promote protein synthesis. Activation of mTORC1 on eIF4B and S6 can promote protein synthesis [36]. Meanwhile, mTORC2 signaling pathway can be activated. mTORC1 regulates cell growth and metabolism, while mTORC2 mainly controls cell proliferation and survival by phosphorylating and activating Akt, which in turn promotes cell proliferation and growth by inhibiting the mTORC1 inhibitor TSC1/2 through phosphorylation [37]. In addition, mTORC2 can activate Rho, PKC, and SGK1 through phosphorylation, thereby regulating cytoskeleton tissue and improving cell survival [38]. Overall, zein peptides play a beneficial effect on C_2_C_12_ cells through MAPK and mTOR/PI3K/Akt pathways, by promoting cytoskeletal protein synthesis and stimulating cell proliferation and survival. In the present study, zein peptides can cause the upregulation of mTORC1 signaling pathway, inhibit the phosphorylation of 4E-BP, and promote the phosphorylation of S6K in C_2_C_12_ cells. The 4E-BP can inhibit eIF4E, while S6K could promote the phosphorylation of eIF4B. The combination of eIF4E and eIF4B jointly accelerated cell cycle progression. After treatment with zein peptides, mTORC2 signaling pathway was inactivated to promote the phosphorylation of PKC, thus activating the regulation of actin cytoskeleton. Taken together, treatment of C_2_C_12_ cells with zein peptides can contribute to accelerating cell cycle progression and promoting protein synthesis of muscle cells through mTORC1 and mTORC2 signaling pathways.

## 5. Conclusions

The present study investigated the proliferation, cell cycle, and anti-apoptotic effects of zein peptides using myoblast C_2_C_12_ cells as a model. It was found that zein peptides had a significant proliferation-promoting effect on C_2_C_12_ cells, which might be achieved by accelerating the cell cycle from G_0_/G_1_ phase to S phase and promoting cell cycle process. Meanwhile, zein peptides also had a preventive and alleviating effect on the apoptosis of C_2_C_12_ cells. LC-MS/MS analysis revealed that zein peptides were abundant in branched-chain amino acids (esp. Leu). Transcriptomics analyses confirmed the beneficial effects of zein peptides on myoblasts through mTORC1 and mTORC2 signaling pathways, promoting cell proliferation and protein synthesis, as well as accelerating cell cycle progression. Overall, zein peptides with a beneficial effect on myoblasts have great potential as a functional ingredient for ameliorating sarcopenia.

## Data Availability

The original contributions presented in the study are included in the article/Supplementary Material, further inquiries can be directed to the corresponding author.

## Supplementary Materials

The following supporting information can be downloaded at: https://www.mdpi.com/article/10.3390/foods13060919/s1, Figure S1: The cluster heat map of differentially expressed genes in zein peptides group, Figure S2: (A) GO annotations of up-regulated genes in zein peptides group, (B) GO annotations of up-regulated genes in zein peptides group, Figure S3: (A) The functionally enriched KEGG pathways based on the up-regulated genes, (B) The functionally enriched KEGG pathways based on the down-regulated genes, Table S1: The representative zein peptide sequences identified by LC-MS/MS.
