# Supplementary material for: Zein-Derived Peptides from Corn Promote the Proliferation of C2C12 Myoblasts via Crosstalk of mTORC1 and mTORC2 Signaling Pathways
Source: Foods. 2024 Mar 18;13(6):919. doi: 10.3390/foods13060919 (PMC10970237; doi:10.3390/foods13060919)
Supplement: Supplementary file 1 [file foods-13-00919-s001.zip › foods-2866266-supplementary.pdf]

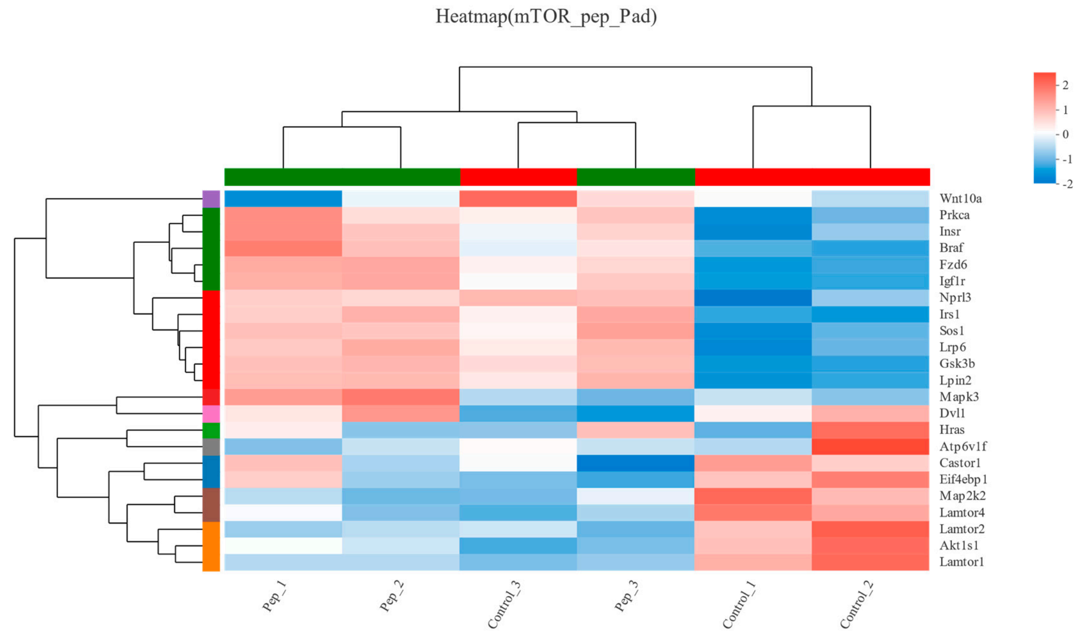

Figure S1 The cluster heat map of differentially expressed genes in zein peptides group.

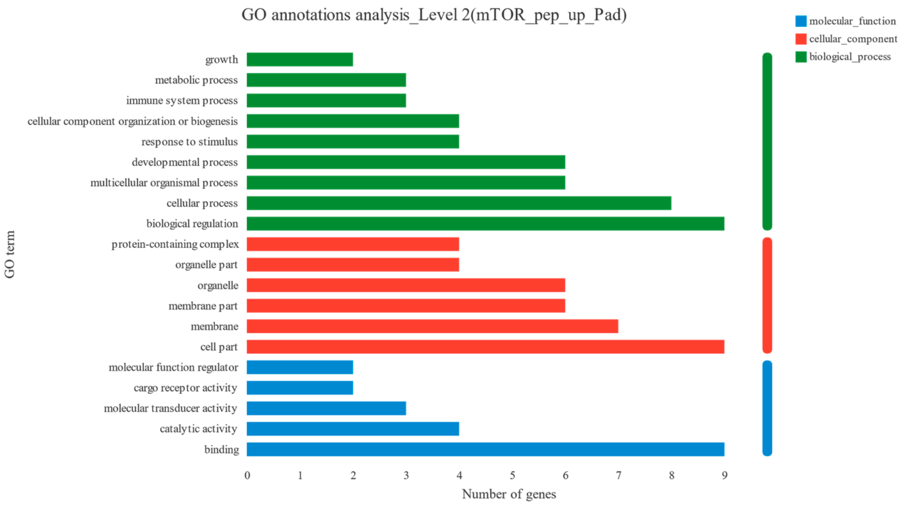

Figure S2A GO annotations of up-regulated genes in zein peptides group.

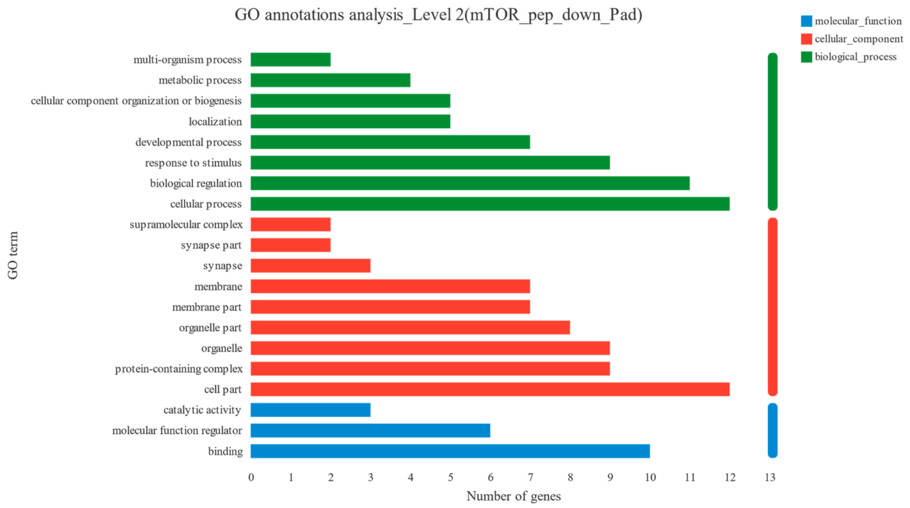

Figure S2B GO annotations of up-regulated genes in zein peptides group.

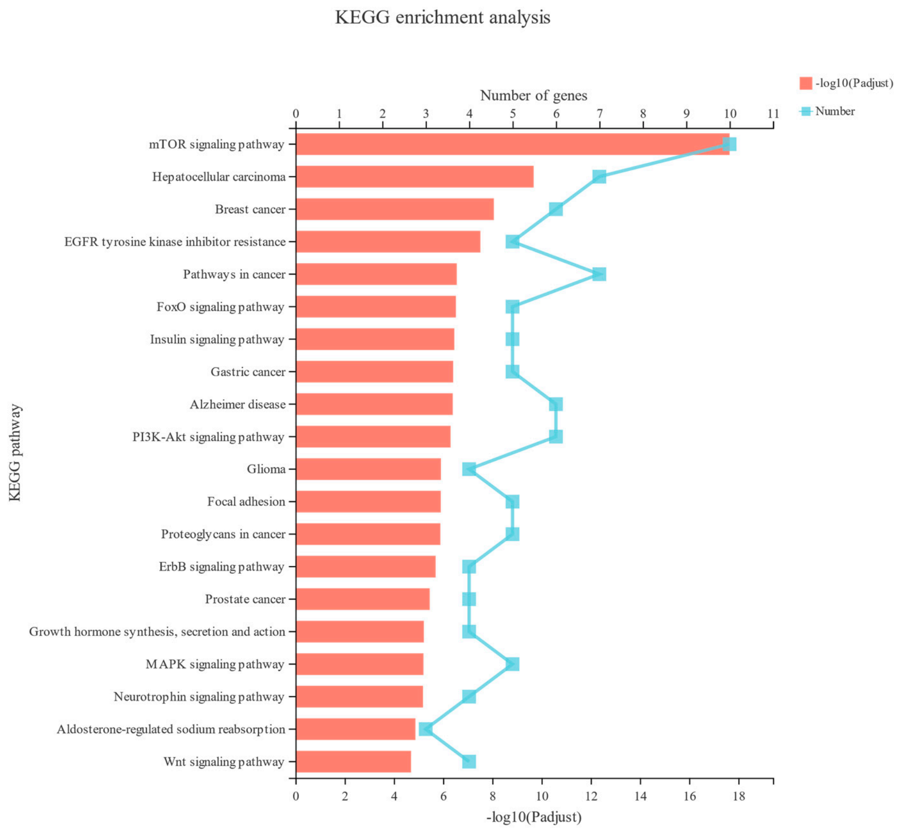

Figure S3A The functionally enriched KEGG pathways based on the up-regulated genes.

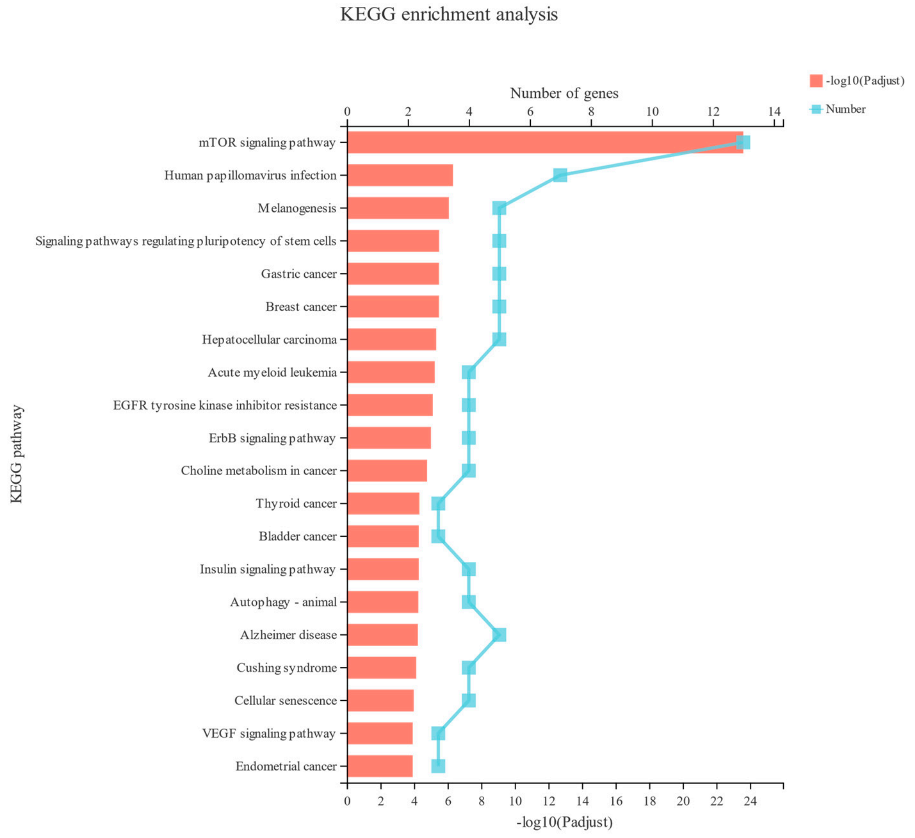

Figure S3B The functionally enriched KEGG pathways based on the down-regulated genes.

Table S1. The representative zein peptide sequences identified by LC-MS/MS

Peptides sequence	Intensity	Precursor protein
**LL**PPY**L**PS	2.06E+11	z1B alpha zein protein
SN**I**P**L**SP**L**	1.67E+11	z1A alpha zein protein
F**L**PPVTSM	1.48E+11	z1C alpha zein protein
S**LL**PPY**L**PS	1.44E+11	z1B alpha zein protein
S**LL**PPY**L**SPA	1.33E+11	Z1A alpha zein protein
ENP**IL**QPY	8.45E+10	Z1A alpha zein protein
**LL**PPY**L**SPA	5.58E+10	Z1A alpha zein protein
YQQP**II**GGA	4.69E+10	prolamin 19 kDa alpha zein z1A1_5 precursor
ENPA**L**QPY	3.87E+10	prolamin 19 kDa alpha zein z1B_6 precursor
GFEHPA**V**Q	3.57E+10	z1C alpha zein protein
RQQ**LL**NP**L**	3.11E+10	22 kDa alpha-zein 14 precursor
RFEYPT**I**Q	2.16E+10	z1D alpha zein protein
YQQP**II**GG	1.67E+10	prolamin 19 kDa alpha zein z1A2_1 precursor
QRQQ**LL**NP**L**	1.66E+10	22 kD alpha-zein
**LL**PPY**L**SP	1.49E+10	Z1A alpha zein protein
AAPNAGT**LL**	1.43E+10	prolamin 19 kDa alpha zein z1A1_7 precursor
**L**PPY**L**SPA	1.41E+10	Z1A alpha zein protein
Q**LL**PFYPQ	1.04E+10	z1D alpha zein protein
ENP**I**VQPY	9.87E+09	prolamin 19 kDa alpha zein z1A2_2 precursor
F**L**RPVTSM	5.33E+09	22-kDa alpha zein 8

Note: The bold front indicates BCAA.
